# Comparative differences in job preferences among doctors in different levels of public hospitals in Henan, China: a discrete choice experiment

**DOI:** 10.3389/fpsyg.2025.1607061

**Published:** 2025-07-23

**Authors:** Jingjing Wang, Hui Lv, Qin Qin, Wenjie Ren, Noorsuzana Mohd Shariff

**Affiliations:** ^1^Institutes of Health Central Plains, Xinxiang Medical University, Xinxiang, Henan, China; ^2^Advanced Medical & Dental Institute, Universiti Sains Malaysia, Kepala Batas, Penang, Malaysia; ^3^The First Affiliated Hospital of Xinxiang Medical University, Xinxiang, Henan, China; ^4^Department of Medical, Quzhou College of Technology, Quzhou, Zhejiang, China

**Keywords:** job posting, job choices, job preference, doctors, healthcare workers

## Abstract

**Background:**

Doctor turnover is a significant challenge faced by hospitals worldwide, particularly in public hospitals in China. By understanding how job attributes influence job preferences among doctors, effective and targeted policies can be designed to attract doctors to key positions or organizations, providing insights for evidence-based workforce interventions.

**Objectives:**

The aim of our study is to examine the difference of job preferences of doctors in different hospitals levels using DCE.

**Methods:**

A discrete choice experiment was applied in the public hospital in China. A total of 920 eligible doctors from primary, secondary, and tertiary public hospital in China participated in this study. A mixed logit model was applied to assess job preferences among doctors in public hospital in China.

**Results:**

Doctors in primary hospitals preferred job offers with 30% salary increase over the unchanged (OR = 8.906, *p* < 0.001). Similar trends were observed in secondary and tertiary hospitals (OR = 4.785, *p* < 0.001; OR = 5.56, *p* < 0.001). Doctors from all hospital levels (primary, secondary, and tertiary) preferred sufficient opportunities of professional development (OR = 1.43, *p* = 0.032; OR = 1.47, *p* = 0.008; OR = 1.44, *p* < 0.001). A 20% increase in workload was generally less favored in all hospital levels (OR = 0.63, *p* = 0.015; OR = 0.45, *p* < 0.001; OR = 0.42, *p* < 0.001). Environmental support was only preferred in tertiary hospitals (OR = 1.46, *p* < 0.001). Monthly salary was the top priority for doctors across all hospital levels, followed by work atmosphere. Employee care came third in primary and secondary hospitals, while opportunities of professional development ranked the third in tertiary hospitals. Environmental support from hospital was the least prioritized factor in both secondary hospitals, while workload was the least important in primary and tertiary hospitals.

**Conclusion:**

Doctors preferred job offers with salary increases across all hospital levels. Professional development opportunities were prioritized universally, while 20% increase in workload was generally least favored. Environmental support was most important in tertiary hospitals. Monthly salary and work atmosphere were top priorities across all hospital levels, with employee care ranking third in primary and secondary hospitals. Workload and environmental support were the least prioritized, varying by hospital level.

## Introduction

The retention and motivation of healthcare workers, particularly public sector doctors, represents a significant challenge across healthcare systems worldwide (de Vries et al., 2023; Zhang et al., 2019). Public sector doctors, as critical providers of medical services, are essential to the functioning and growth of hospitals, with their self-made decisions regarding retention and turnover directly influencing efficiency, capacity expansion, service quality, and the support of research and development efforts. A key issue facing healthcare systems globally is the inadequate and unequal distribution of health professionals, which exacerbates the challenges related to workforce sustainability (Berman et al., 2021). In 2023, The Lancet Global Health reported that the healthcare sector faced a critical shortage of workers (The Lancet Global Health, 2023). A recent report from the Pan American Health Organization (PAHO) further highlights this issue, indicating that 14 out of 39 countries in the Americas lack an adequate number of healthcare workers, including doctors, nurses, and midwives (Pan American Health Organization, 2025). The report also projects that, without immediate interventions, the Americas could face a deficit of between 600,000 and 2 million healthcare workers by 2030.

Studies consistently showed that healthcare worker turnover, particularly among doctors, was a key factor leading to staff shortages (Koch et al., 2020; Kraut, 1975). Given the challenges in tracking and measuring turnover behaviors in real-world settings, Kraut (1975) firstly proposed that employee turnover could be predicted through turnover intention. Studies have consistently highlighted the high prevalence of turnover intentions among healthcare workers worldwide. For example, in Ethiopia, 67.8% of health workers in primary hospitals expressed the intention to leave due to job dissatisfaction and low remuneration (Worku et al., 2019). Similarly, 17% of physicians in Europe planned to leave their hospitals, with 9% considering leaving the profession entirely (Maniscalco et al., 2024). A survey in Iraq found an alarming 85% turnover intention rate (Al-Mousawi and Lafta, 2024), while in China, 74.8% of resident physicians showed similar intentions (Jia et al., 2023). Key factors contributing to turnover include limited career development opportunities (Tekle et al., 2022), job burnout (Jia et al., 2023; Ran et al., 2020; Chen et al., 2022), poor working conditions (Scott et al., 2013), low job satisfaction (Ran et al., 2020; Ning et al., 2023), high workload (Tekle et al., 2022), job stress (Ning et al., 2023), and low incentives (Tekle et al., 2022). Public hospitals, in particular, present additional challenges such as low pay, heavy workloads, and heightened patient expectations, all of which increase job dissatisfaction and burnout. In contrast, private hospitals typically offer better working conditions, higher pay, and lower workloads, potentially reducing turnover rates. For instance, a study in Cyprus found that 49.8% of physicians in public hospitals had turnover intentions (Gregoriou et al., 2023), while a comparison in Canada showed 34.2% in both public and private hospitals (Sheekha et al., 2024). These findings underscore the significant impact of hospital environment on turnover decisions. Job dissatisfaction often precedes high turnover, which in turn exacerbates the shortage of healthcare professionals, particularly in countries like China. Understanding these factors is crucial for hospital administrators to design effective interventions that improve job satisfaction and reduce turnover.

Job preference is a complex decision shaped by a variety of work-related factors (e.g., job characteristics, personal and family-related circumstances) (Fields et al., 2018), and it can vary significantly across countries and hospital types. Job preferences can be categorized into revealed and stated preferences (Samuelson, 1938). Revealed job preferences are typically derived from large cohort databases in empirical research, which gather data on the factors influencing individuals’ decisions to accept or leave jobs (Louviere and Lancsar, 2009). This type of evidence, based on administrative or observational data, is commonly analyzed using econometric techniques to explore explicit job preferences (Antonazzo et al., 2003; Lagarde and Blaauw, 2009). However, obtaining reliable and accurate data in practice proves challenging, as these databases often require extensive longitudinal information, which may not always be available (Lagarde and Blaauw, 2009). This limitation underscores the difficulty in capturing true behavioral preferences in real-world settings. In contrast, Stated Preference techniques, such as surveys and discrete choice experiments (DCEs), have been widely employed to assess individuals’ preferences and their willingness to engage in specific healthcare-related activities, especially in the field of health economics (de Bekker-Grob et al., 2020).

Traditional survey methods, such as questionnaires and interviews, often struggle to capture job preferences due to the complexity of combining multiple related factors. In contrast, Discrete Choice Experiments (DCEs) are a distinct survey-based approach that allows for a more precise assessment of preferences by presenting respondents with hypothetical choices based on specific attributes (Liu et al., 2019). As a stated preference survey method, discrete choice experiment (DCE) is used to quantify the relative importance of factors influencing decision-making (Fields et al., 2018) and to provide a choice-based measure of benefit (Abdelhadi et al., 2023). The targeted participants could represent their preferences for hypothetical alternatives defined by various attributes (e.g., job characteristics) and their corresponding levels (Cleland et al., 2022). DCEs have gained prominence in health economics due to their ease of implementation, efficiency, and ability to generate data that address critical questions over the past two decades, particularly in studies exploring the job preferences of health professionals (de Bekker-Grob et al., 2020; Cleland et al., 2022; Ejebu et al., 2024). This method is based on McFadden’s (1973) random utility theory, which links deterministic utility (derived from observable attributes and levels) to choice probabilities (Rheindorf et al., 2024). Respondents select their preferred alternative from a set of options in a discrete choice experiment, and these choices are used to infer preferences for all attribute levels. This approach aligns with the majority of Discrete Choice Experiments (DCEs) in health economic, where studies typically included two to six attributes, and each respondent was asked to make 16–20 choices (Tozduman and Sözmen, 2024).

Existing studies have identified key factors influencing job preference using discrete choice experiments (DCE). Nevertheless, the variations in the research subjects have contributed to differences in the results observed. Zhang et al. found that monthly income and work location were the most salient factors for medical students when considering choosing jobs (Zhang et al., 2023). Bao et al. reported that “Bianzhi” (the number of personnel allocated to each employer by the government) and physical conflicts between doctors and patients were two of the most important non-monetary job characteristics for medical and nursing students (Bao and Huang, 2021). These attributes reflect critical factors within the Chinese healthcare environment. ‘Bianzhi’ pertains to job stability, a characteristic highly valued by Chinese doctors, while physical conflicts between doctors and patients underscore concerns related to workplace safety and interpersonal dynamics. Mumbauer et al. found that worker preferences were significantly influenced by heavy workload, poor workplace culture, insufficient availability of equipment and infrequent training opportunities (Mumbauer et al., 2021). A study conducted in China identified that primary care providers value highly monetary benefits, availability of equipment and respect from the community (Song et al., 2015). Scott et al. suggested that while physicians generally preferred the public sector, their preferences were shaped by factors such as risk aversion, earnings, and career risks, with lower earners and those averse to risk favoring the public sector, while higher earners tended to prefer the private sector (Scott et al., 2020). Pestana et al. indicated that physicians strongly preferred jobs offering more autonomy and training opportunities (Pestana et al., 2024). Matiwane et al. reported that doctors strongly opposed banning multiple job holding, requiring a 45.7% salary increase to accept the ban, were willing to forgo 57.9% of their salary for an improved clinical practice environment, and valued competent hospital management (Matiwane et al., 2025). Tozduman and Sözmen found that monetary incentives were the most effective for recruiting young physicians, while non-pecuniary factors and individual characteristics also played a significant role in shaping job preferences (Tozduman and Sözmen, 2024).

Although discrete choice experiments (DCEs) have been widely used in international studies to examine job preferences among healthcare workers, fewer studies have addressed job preferences among doctors across different levels of public hospitals. This study contributes to the literature by exploring the job preferences of doctors at primary, secondary, and tertiary public hospital levels, thereby addressing this gap and providing valuable insights into doctor-specific job determinants. Therefore, our research aim was as follows: (1) The research question was “What are the core job attributes preferred by doctors in different hospitals levels?.” (2) The heterogeneity question was “What are the differences in job preferences among doctors working in primary, secondary, and tertiary public hospitals?” (3) The hypothesis was “There were differences in the job preferences of doctors in different hospitals levels.”

## Methods

### Survey design

A discrete choice experiment was used to design the questionnaire. Discrete choice experiments provide a more nuanced examination of the trade-offs between different attributes, offering greater insight than traditional regression analyses (Chudner et al., 2019). This multi-step process—including literature review, stakeholder engagement, interviews, and pilot testing—ensured the robustness, relevance, and credibility of the DCE design. In the initial phase, a comprehensive literature review was conducted to identify the key job attributes relevant to job preference among doctors, and it has been shown in Appendix 1. Following this, focus group discussions with a panel of experts, including hospital administrators and healthcare professors, were held to refine and confirm the relevance of the attributes and their associated levels within the Chinese healthcare context. Semi-structured interviews with a diverse sample of senior doctors in public hospitals were then conducted to assess the feasibility and relevance of the identified attributes in real-world settings and to gain insights into the most critical job attributes and their specific levels. Subsequently, a pilot test was conducted with a small group of senior doctors to assess the clarity, relevance, and appropriateness of the attributes and levels in the DCE questionnaire. Feedback from this testing led to refinements in the language and presentation of certain attributes, ensuring their comprehensibility and applicability. Following these steps, the data were refined into six key attributes, each defined by two to three levels (Table 1). The hypothetical job scenarios created for the DCE were designed to reflect job options within public hospitals, ensuring the experiment’s realism and practical applicability.

**Table 1:** Discrete choice experiment attributes and levels.

Attributes	Levels	Definitions
Monthly salary	Decreased 30%, unchanged; increased 30%	
Opportunities of professional development	Limited; general; sufficient	Including training opportunities, career promotion
Workload	Decreased 20%, unchanged; increased 20%	Work hours every week
Environment support from hospitals	insufficient, sufficient	Including security, technological equipment support
Employee care	insufficient, sufficient	Including welfare, the respect from leader
Work atmosphere	Unsupportive, Supportive	Including teamwork, support from team

After identifying the attributes and levels, the hypothetical choice sets were created by Stata software. With six attributes, three with three levels and three with two levels, our study included a total of 216 (2^3^ × 3^3^) possible scenarios. This would result in 23,220 ((216 × 215)/2) choice sets, with two options included in each choice set. If presenting all of these scenarios to each doctor using a full factorial design, it is difficult for doctors to complete it due to the high cognitive burden. Therefore, we applied a D-efficient Bayesian design to achieve with 16 pairs of choice sets to implement this study. This design utilized a full-profile, conjoint choice approach with balanced overlap, aiming to minimize the number of choice sets presented to respondents while ensuring a comprehensive representation of the attribute levels (Johnson et al., 2013). Stata software was employed to generate a fractional factorial design, which divided the survey into two versions (Version A and Version B), each containing eight choice sets. Additionally, we added a choice set to assess the respondent’s seriousness in completing the questionnaire, ensuring its validity (Wu et al., 2021). The choice set consists of a worst-case and a best-case job scenario. If the respondent selects the worst-case job, the questionnaire is considered invalid. This is also helpful for accessing the internal consistency of the measure and the rationality of participants. As a result, each vision had nine choice sets in our study.

**Figure 1:**
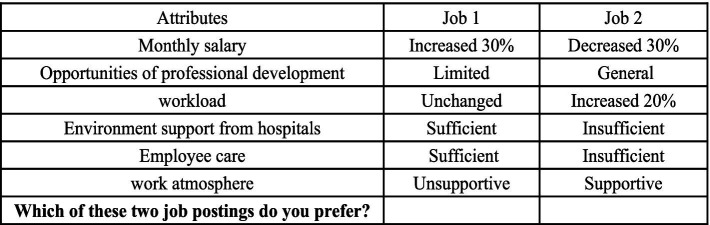
Example choice set: doctors.

Two job scenarios were presented in each choice set to the targeted doctors, who were asked to select their preferred option. Each alternative was defined by a distinct set of attributes and corresponding attribute levels. The questionnaire explicitly stated that there were no right or wrong answers, instructing respondents to base their choices on their actual situation to ensure the research’s rigor. An example of a choice set was shown in Figure 1. The study also incorporated several demographic questions, including age, sex, marital status, education level, and years of experience.

### Survey sample and method

Henan, a large and populous province in central China, features a diverse healthcare system composed of various types of hospitals, including tertiary, secondary, and primary-level institutions. Tertiary hospitals are typically large, comprehensive medical centers equipped with advanced technologies and specialized medical services. In contrast, secondary hospitals provide more general medical care, while primary hospitals offer basic healthcare services, particularly to rural populations. Participants in this study were drawn from four tertiary hospitals, four secondary hospitals, and fifteen primary hospitals, resulting in a total of 23 public hospitals and 977 participating doctors. Pearmain (1991) recommended that a minimum sample size of 100 is necessary to conduct a DCE study effectively. Orme proposed the formula for calculating the minimum sample size: N > 500c/(t*a) (Orme, 2010). In this formula, where “c” represents the maximum number of levels for the attributes, “t” represents the number of choices sets, “a” means the number of options for each choice set. In this study, c = 3; t = 9, a = 2, 500c/(t*a) = 500*3/(9*2) = 83.3. Data were collected between July 2024 and February 2025 through both face-to-face and online surveys. We established specific inclusion and exclusion criteria to identify the targeted doctors. The inclusion criteria were as follows: (1) possessing a professional doctors’ certificate (2) working for 6 months or greater (3) being volunteered to participate in the survey. The exclusion criteria were as follows: (1) doctors who were unavailable to participate due to time constraints, or other personal reasons (2) doctors who were engaged in continuing education, on maternity leave, or on sick leave. Specifically, participants could participate in this survey by scanning a code or receiving a paper questionnaire. In addition, a designated contact person was assigned at each sample hospital to assist with coordination to ensure the smooth execution of the study. A small gifts or red envelopes were provided to doctors who completed the questionnaire to express our gratitude.

### Ethical considerations

The study protocol was approved by the Ethics Committee of the Universiti Sains Malaysia (approval number USM/JEPeM/PP/24060491). Doctors were provided a full explanation of the study’s aims, including their right to quit, and assured of confidentiality.

### Data analysis

Demographic information was analyzed using univariate, descriptive statistics (e.g., percentages). A mixed logit model was utilized to examine doctors’ preferences for the different levels of the job attributes. The mixed logit model accounts for preference heterogeneity within the sample by modeling the coefficients as random (Fields et al., 2018). Main effects were presented in all models in our study, without interaction terms. All attributes were dummy coded and specified as having a random component. Simulated maximum likelihood with 500 Monte Carlo was utilized to estimate all the models due to the non-closed form (Hilo and McPeak, 2024). Subsequently, the relative importance (RI) of each attribute was calculated by summing the utility differences between the maximum and minimum regression coefficients within a specific attribute, and then dividing this sum by the total utility difference across all attributes in the model (Determann et al., 2016). The formula is as follows:


$$\mathrm{RIk}=\frac{\max\beta_{k}-\min\beta_{k}}{\sum_{1}^{k}(\max\beta_{k}-\min\beta_{k})}$$

Additionally, the regression coefficients of six attributes from the mixed logit model were utilized to predict the doctors’ job preferences, assuming that each job attribute changed by one level while holding the levels of other job attributes constant. One of the key advantages of discrete choice experiment (DCE) analysis is its capacity to assess the change in the probability of selecting a baseline attribute when the level of a specific attribute is altered (Wu et al., 2021). This serves as an effective predictor for hospital managers to assess the effect of changes in job attributes. For example, if one job option offers a 30% salary decrease and the other offers a 30% salary increase, the likelihood of doctors choosing the job with the 30% salary increase will increase. The formula is as follows:


$$\begin{array}{l} P=P30\%\text{increase in salary}-P30\%\text{decrease in salary}\\ =\frac{e^{\beta 2}}{e^{\beta 1}+e^{\beta 2}}-\frac{e^{\beta 1}}{e^{\beta 1}+e^{\beta 2.}} \end{array}$$

All analyses were conducted using Stata version 17 with significance at 0.05.

## Results

Out of the 1,026 doctors approached, 977 responses were collected, resulting in a response rate of 95.2%. Fifty seven doctors selected the worst job scenario from a choice set combining both the best and worst scenarios, resulting in the exclusion of this data as invalid, and thus 920 eligible doctors were included for analysis.

### Sample characteristics

Table 2 presents the general characteristics of the surveyed doctors working in the primary, secondary, and tertiary hospitals. The highest proportion of doctors (51.41%) was from tertiary hospitals, followed by those from secondary hospitals (25.98%). The distribution of sex was fairly balanced, with 44.35% of the participating doctors being male. The highest proportion of male doctors was observed in tertiary hospitals (50.98%). 48.37% of the doctors were aged between 30 and 40 years, followed by 22.07% in the 40–50 age range. Notably, doctors under 30 years were most prevalent in primary hospitals (68.39%), while doctors aged 30–40 were most represented in tertiary hospitals (56.63%). The majority of doctors (78.70%) were being married, and primary hospitals had the highest proportion of single doctors (70.92%). 63.46% of the doctors held a bachelor’s degree, and only 10.44% had an associate degree or less. These doctors with master’s or doctorate degrees accounted for the highest proportion (93.33%) in tertiary hospitals, while those with an associate degree or less had the highest proportion (84.31%) in primary hospitals. 48.15% of the doctors had at least 10 years of professional experience. In primary hospitals, 69.81% of doctors had 5 years or less of experience, whereas in tertiary hospitals, 37.25% had more than 10 years of experience.

**Table 2:** General characteristics of doctors.

Characteristics		*N* (%)	Primary hospital (CHS and TH)	Secondary hospital	Tertiary hospital
		920	208 (22.61)	239 (25.98)	473 (51.41)
Sex	Male	408 (44.35)	91 (22.30)	109 (26.72)	208 (50.98|)
	Female	512 (55.65)	117 (22.85)	130 (25.39)	265 (51.76)
Age	less than 30	174 (18.91)	16 (9.20)	39 (22.41)	119 (68.391)
	30–40	445 (48.37)	81 (18.20)	112 (25.17)	252 (56.63)
	40–50	203 (22.07)	70 (34.48)	56 (27.59)	77(37.93)
	more than 50	98 (10.65)	41 (41.84)	32 (32.65)	25 (25.51)
Marital status	Single(Unmarried/ Divorced/Widowed)	196(21.30)	20 (10.20)	37 (18.88)	139 (70.92)
	Married	724(78.70)	188 (25.97)	202 (27.90)	334 (46.13)
Education level	Associates degree or less	102 (10.44)	86 (84.31)	12 (11.76)	4 (3.92)
	Bachelor Degree	620 (63.46)	136 (21.94)	229 (36.94)	255 (41.13)
	Masters or Doctorate Degree	255 (26.10)	2 (0.78)	15 (5.88)	238 (93.33)
Years of experience	5 years or less	265 (28.81)	21 (7.92)	59 (22.26)	185 (69.81)
	5–10 years	212 (23.04)	35 (16.51)	54 (25.47)	123 (58.02)
	10 years and more	443 (48.15)	152 (34.31)	126 (28.44)	165 (37.25)

### Heterogeneity of job preferences among doctors under hospital types

The results of the mixed logit model revealed significant preferences among doctors for various work attributes, with considerable variations across hospitals levels (Table 3). The model provided robust goodness-of-fit statistics, with the likelihood ratio chi-squared statistic being significant (*p* < 0.0001), indicating a well-fitting model for all hospital levels. The log-likelihood values for primary, secondary, and tertiary hospitals were −723.93, −772.45, and −1553.54, respectively.

**Table 3:** Estimation of mixed logit model for job preferences among doctors.

Attributes	Primary hospital		Secondary hospital		Tertiary hospital	
	OR(95% CI)	*p*	OR(95% CI)	*p*	OR(95% CI)	*p*
Monthly salary						
Unchanged (ref)						
Decreased 30%	0.17 (0.064, 0.283)	<0.001	0.14 (0.056, 0.029)	<0.001	0.16 (0.100,0.227)	<0.001
Increased 30%	8.91 (3.163, 14.649)	<0.001	4.79 (2.643, 6.927)	<0.001	5.56 (3.841,7.277)	<0.001
Opportunities of professional development						
Limited (ref)						
General	0.74 (0.483, 1.000)	0.093	0.87 (0.610, 1.123)	0.342	0.78 (0.619, 0.938)	0.017
Sufficient	1.43 (0.961, 1.904)	0.032	1.47 (1.051, 1.881)	0.008	1.44 (1.155, 1.721)	<0.001
Workload						
Decreased 20% (ref)						
Unchanged	0.63 (0.421, 0.841)	0.007	0.64 (0.434, 0.853)	0.008	0.58 (0.469, 0.693)	<0.001
Increased 20%	0.63 (0.391, 0.863)	0.015	0.45 (0.288, 0.610)	<0.001	0.42 (0.318, 0.515)	<0.001
Environment support from hospitals						
Insufficient (ref)						
Sufficient	1.14 (0.829, 1.455)	0.342	1.20 (0.917, 1.476)	0.132	1.46 (1.236, 1.676)	<0.001
Employee care						
Insufficient (ref)						
Sufficient	1.99 (1.424,2.552)	<0.001	2.36 (1.747,2.973)	<0.001	1.78 (1.504,2.062)	<0.001
Work atmosphere						
Unsupportive(ref)						
Supportive	2.83 (1.813, 3.842)	<0.001	3.17 (2.232, 4.104)	<0.001	3.62 (2.771, 4.474)	<0.001
*N*	920		920		920	
Observation	3,328		3,824		7,568	
Log likelihood	−723.93		−772.45		−1553.54	
LR χ2	174.43		130.21		218.74	
Prob > χ2	< 0.0001		< 0.0001		< 0.0001	

Our results showed that doctors in primary hospitals preferred job offers with either a 30% increase in salary, as opposed to or an unchanged salary (OR = 8.91, *p* < 0.001). Similar trends were observed for doctors in secondary and tertiary hospitals (OR = 4.79, *p* < 0.001, OR = 5.56, *p* < 0.001). We also found that doctors in different hospitals levels did not like a 30% decrease in salary (OR = 0.17, *p* < 0.001, OR = 0.14, *p* < 0.001, OR = 0.16, *p* < 0.001). In terms of professional development opportunities, compared to limited opportunities, general opportunities did not show significant preference in primary and secondary hospitals (OR = 0.74, *p* = 0.093; OR = 0.87, *p* = 0.342), while a significant preference was observed in tertiary hospitals (OR = 0.78, *p* = 0.017). Doctors across all hospital levels strongly preferred sufficient opportunities for professional development (OR = 1.43, *p* = 0.032; OR = 1.47, *p* = 0.008; OR = 1.44, *p* < 0.001). Regarding workload, an unchanged workload compared to a 20% decrease was less favored across all hospital types (OR = 0.63, *p* = 0.007; OR = 0.64, *p* = 0.008; OR = 0.58, *p* < 0.001), indicating a lower preference for this attribute level. Similarly, a 20% increase in workload was associated with a decreased preference for job posts across all hospital types (OR = 0.63, *p* = 0.015, OR = 0.45, *p* < 0.001; OR = 0.42, *p* < 0.001), suggesting that higher workloads reduce preference for the positions. As for environmental support from hospitals, sufficient environment support from hospitals was only favored in tertiary hospitals (OR = 1.46, *p* < 0.001), and no significant preferences were found in primary hospitals (OR = 1.14, *p* = 0.342) or secondary hospitals (OR = 1.20, *p* = 0.132). Regarding work atmosphere, a supportive work atmosphere was associated with an increased preference for job posts across all hospital types (OR = 2.83, *p* < 0.001, OR = 3.17, *p* < 0.001; OR = 3.62, *p* < 0.001).

### Relative importance of job preferences among doctors under hospital types

**Figure 2:**
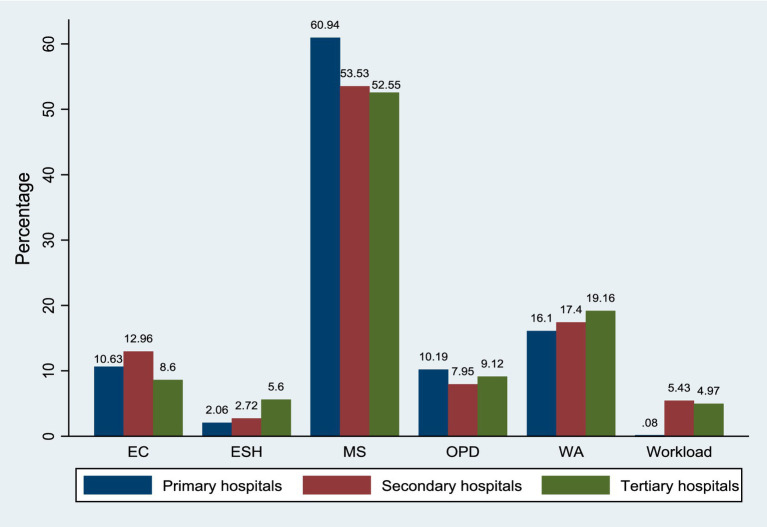
Percentage of relative importance of work attributes among doctors under hospital types. EC = Employee care; ESH = Environment support from hospitals; MS = Monthly salary; OPD=Opportunities of professional development; WA = Work atmosphere.

The relative importance of various work attributes among doctors across different hospital types was depicted in Figure 2. Monthly salary emerged as the most significant factor for doctors at all hospital levels, with primary hospitals assigning the highest importance (60.94%). Work atmosphere also played a crucial role in doctors’ job preferences, with the highest weight assigned by tertiary hospitals (19.16%). Employee care was of moderate importance across all hospital types, with primary hospitals at 10.63%, secondary hospitals at 12.96%, and tertiary hospitals assigning the least importance at 8.60%. Opportunities for professional development were considered by doctors but were relatively less significant compared to other attributes. Workload was the least important factor across all hospital types, with least weight assigned in primary hospitals (0.08%), followed by secondary hospitals (5.43%) and tertiary hospitals (4.97%). Finally, environmental support from hospitals emerged as another lower-priority attribute across all hospital levels.

### Predictive analysis of job preference under different condition interventions

Table 4 demonstrates that, with a baseline of the unchanged in monthly salary, limited career development opportunities, 20% reduction in workload, insufficient hospital support from hospitals, insufficient employee care, and unsupportive work atmosphere, when salary was replaced with a 30% increase in salary levels, the probability of doctors’ choice increased across all hospital levels, with primary hospitals showing the largest increase (79.82%), followed by tertiary hospitals (69.50%). In contrast, when salary was replaced with a 30% decrease in salary levels, the probability of doctors’ choices decreased across all hospital levels, with secondary hospitals showing the largest decrease (75.78%), followed by tertiary hospitals (71.92%). When career development opportunities were adjusted to a general level, job choice probability decreases only for doctors in tertiary hospitals (−12.44%). Conversely, when career development opportunities were sufficient, choice probability of doctors increased across all hospital levels, with the greatest effect in secondary hospitals (18.92%), followed by tertiary hospitals (17.95%). When workload remains constant, job choice probability declined at all hospital levels, with the largest decrease observed in tertiary hospitals (26.50%). A 20% increase in workload led to the greatest reduction in tertiary hospitals (41.24%). With sufficient environmental support, choice probability raised by 18.58%, but this effect is only significant for doctors in tertiary hospitals. Additionally, sufficient employee cares most impacted secondary hospitals (40.49%), while tertiary hospitals experienced the smallest increase (28.12%). Finally, supportive work atmosphere increased job choice probability at all hospital levels, with the largest effect in tertiary hospitals (56.73%).

**Table 4:** Predictive analysis of job preference under different condition interventions.

Properties		Primary hospitals	Secondary hospitals	Tertiary hospitals
		(%)	(%)	(%)
Monthly salary	Unchanged (ref)			
	Decreased 30%	−70.39	−75.78	−71.92
	Increased 30%	79.82	65.41	−69.50
Opportunities of professional development	Limited (ref)			
	General			−12.44
	Sufficient	17.76	18.92	17.95
Workload	Decreased 20% (ref)			
	Unchanged	−22.65	−21.70	−26.50
	Increased 20%	−22.89	−38.04	−41.24
Environment support from hospitals	Insufficient (ref)			
	Sufficient			18.58
Employee care	Insufficient (ref)			
	Sufficient	33.06	40.49	28.12
Work atmosphere	Unsupportive (ref)			
	Supportive	47.78	52.01	56.73

## Discussion

To our knowledge, this is the first DCE to be conducted with doctors from different levels of public hospital in China to investigate doctors’ job preferences, revealing that doctors in different hospitals levels exhibited both consistency and variability in their preferences for work attributes. This means that it can provide different hospitals levels with preliminary information on which job characteristics matter most to doctors in public hospital in China. Our findings indicated that monthly salary, work atmosphere, employee care and opportunities of professional development had the significant effect on the job choices of doctors in different hospital levels; however, only doctors from tertiary hospitals place significant value on environmental support.

Firstly, our results indicated that doctors in different levels of hospitals consistently prefer job offers involving a salary increase, which emerged as the most important attribute for doctors. This indicates that salary remains a dominant consideration for doctors in all settings, though it holds slightly more importance for those in primary hospitals. This finding aligns with the job preferences of primary physicians in China (Bao et al., 2023; Wang et al., 2024), Malawi (Berman et al., 2021), Ethiopia (Hilo and McPeak, 2024), Türkiye (Islek and Sahin, 2023) and Uganda (Law et al., 2021), highlighting that increasing salary could potentially deal with the shortages of health worker. However, other studies conducted in other countries got the opposite results (Gadsden et al., 2022; Kruk et al., 2010)^.^, and it could be explained by the difference on the background of labor market. Similar to community health workers in Indonesia, who preferred lower monthly financial benefits, this preference arises from the kader program, which trains local volunteers in rural areas to provide basic health services and education, grounded in the cultural and religious value of gotong royong (Gadsden et al., 2022). This concept promotes cooperation, responsibility, and community solidarity, encouraging individuals to contribute to the community without expecting high monetary rewards (Suwignyo, 2019). Additionally, the results of the percentage of relative importance also indicated that monthly salary had the highest weight, particularly in primary hospitals. In China, doctors’ salary in primary hospitals was significantly lower than that of their counterparts in secondary or tertiary hospitals, which makes salary a more influential factor in their job choice decisions. A previous study focusing on doctors in primary hospital of China also proved that salary increases had the most significant effect on physician retention (Bao et al., 2023). Another study indicated that a fourfold increase in salary would be necessary to achieve a 100% probability of physicians selecting rural areas (Islek and Sahin, 2023). Additionally, our attribute prediction analysis revealed that when the monthly salary was replaced to a higher level, the likelihood of doctors in primary hospitals accepting job offers showed the greatest increase.

Secondly, the findings revealed that supportive work atmosphere were preferred by the doctors in all hospital levels. This finding is consistent with the previous study by Li et al., which indicated that the work environment, including superior interpersonal relationships and organizational culture, was the most important non-financial incentive (Li et al., 2024). This attribute was the second most important attribute in all hospital levels, suggesting that improving work atmosphere alongside more attractive factors to attract doctors to stay. The work atmosphere, as defined in our study, primarily encompasses teamwork, support from team. This could be explained by the fact that a supportive work environment contributes to alleviate the work-related stress or burnout experienced by doctor. Additionally, it plays a crucial role in shaping organizational culture and fostering cohesion, which, in turn enhances doctors’ sense of belonging and reduces doctors’ intentions to leave.

Thirdly, our result revealed that employee care was highly valued by doctors in all hospital levels. This attribute ranks fourth in tertiary hospitals, while third in both primary and secondary hospitals. Maslow’s hierarchy of needs theory suggests that once basic material needs are met, individuals are driven to fulfill higher psychological needs, such as employee care in our study. Doctors in higher-level hospitals earn significantly higher salaries, which leads them to place greater emphasis on employee care when considering their work. We found doctors in primary and secondary hospitals also prioritize non-financial incentives. Previous studies have found that non-financial incentives play an important role in job preferences among doctors. İşlek and Şahin indicated that those prioritizing location over wages are likely to favor urban areas that offer advanced healthcare, quality education for children, and social activities (Islek and Sahin, 2023). Angell et al. highlighted the value of non-financial incentives, suggesting that interventions designed to increase community support for doctors and protect them from violence should be implemented (Angell et al., 2021). The previous also indicated that primary care providers value highly welfare (monetary) benefits (Yan et al., 2014). Therefore, when designing intervention strategies, taking into account the diverse needs of doctors from different backgrounds is crucial (Fields et al., 2018).

Fourthly, our findings indicated that opportunities of professional development were also considered by doctors in all hospital levels, though they were ranked less significantly compared to other factors. This is consistent with the finding by Berman et al., revealing that career development opportunities have a limited impact on the job choices of doctors in primary hospitals (Berman et al., 2021). Li et al. also emphasized the strong association between adequate career development and job satisfaction among healthcare workers, particularly when their income reaches a higher level (Li et al., 2024). Similarly, Thai et al. identified career opportunities as the third most important attribute in primary care settings, noting that a lack of professional development could lead to higher rates of employee turnover (Thai et al., 2024). Our result found that the importance of career development opportunities varies across different levels of hospitals when considering job offers. Specifically, career development ranked as the third most important factor for doctors in tertiary hospitals, whereas it was the fourth most important for doctors in primary and secondary hospitals. These findings provide valuable insights for hospital administrators, suggesting the need for tailored measures to motivate doctors at different levels of healthcare institutions. Bao et al. mentioned that government should consider offering preferential access to training in primary hospitals (Bao et al., 2023). This aspiration for professional development opportunities likely stems from the access to prestigious positions and salary increases, as doctors often associate career growth with upward mobility within the healthcare sector.

Additionally, workload was strongly disliked by doctors in all hospital levels, with the second-to-last most important factor in secondary hospitals, and the least important factor in primary and tertiary hospitals. A study conducted in Chinese primary hospitals found that physicians preferred fewer working hours (Bao et al., 2023), which aligns with our findings. Similarly, a study form UK indicated that work hours was the least important attribute to hospital doctors (Cleland et al., 2022), which is line with our current study. However, the study in China focusing on healthcare administration students got the opposite result (Liu et al., 2019), suggesting that perceptions of workload may vary depending on the context. The varying importance of workload across different hospital levels can be attributed to the differing work environments. Doctors in secondary and tertiary hospitals often face significant workloads, and further increases may contribute to burnout due to work overload. In contrast, doctors in primary hospitals typically encounter relatively lower work demands, making workload a less critical factor in career decision-making. Additionally, other studies have highlighted the mismatch between higher workload and income, which plays a significant role in lower job satisfaction among doctors in primary hospitals (Jin et al., 2019). Joyce et al. also suggested that reducing working hours was an effective strategy for retaining doctors, thus helping to extend their careers in hospitals (Joyce et al., 2015). These findings suggest that increasing workload without commensurate rewards or support could lead to higher turnover rates among doctors.

In additionally, we also found that only doctors in tertiary hospitals valued environmental support from the hospital, while no such significant influence was observed on the job choices of doctors in primary and secondary hospitals. This means that doctors in tertiary hospitals are more sensitive to environmental support, exhibiting a stronger preference for hospital facilities and equipment. This could be explained by the fact that insufficient environment support from hospitals likely restricts doctors’ capacity to deliver effective patient care and readiness to practice (Kurniati et al., 2024). The results from Berman et al. also highlighted that job choice of doctors could be impacted by the facility quality (Berman et al., 2021). Doctors in tertiary hospitals typically possess higher education levels, advanced professional skills, and enhanced research capabilities, all of which necessitate robust support from the hospital’s technical infrastructure to be realized. In contrast, doctors in primary hospitals tend to focus on treating common illnesses, which means their demand for advanced environmental support is comparatively lower. This difference in the value placed on environmental support can be explained by the varying levels of specialization and technical requirements across hospital types.

### Strengths and limitations

This study examined differences in doctors’ job preferences across various hospitals levels using a Discrete Choice Experiment (DCE), providing offering valuable insights into the nuanced decision-making processes and key job attributes that influence their preferences. The study successfully reached a large sample of doctors across different hospitals levels, achieving a sufficiently high response rate to allow for robust statistical analysis. However, there were some limitations. Firstly, the use of structured scenarios in the DCE may restrict the generalizability of the findings to real-world situations, as the DCE may not fully capture the complexities of actual decision-making processes. Secondly, the counterfactual simulation used in the study is based on an experimental framework that cannot fully reflect reality, which limits its ability to accurately represent real-life decision-making. Thirdly, the constraints on the number of attributes in the DCE limited the inclusion of additional variables, consequently restricting the ability to capture the full range of factors that may influence work preferences. Additionally, although surveyors provided explanations of the research design, including attributes and levels, to the respondents during the survey and allowed them to complete the questionnaire independently, individual differences in interpretation may still influence the results

## Conclusion

To our knowledge, this is the first DCE to be conducted with doctors from different levels of public hospital in China to investigate doctors’ job preferences, revealing that doctors in different hospitals levels exhibited both consistency and variability in their preferences for work attributes. Our findings indicated that monthly salary, work atmosphere, employee care and opportunities of professional development had the significant effect on the job choices of doctors in different hospital levels; however, only doctors from tertiary hospitals place significant value on environmental support.

## Data Availability

The raw data supporting the conclusions of this article will be made available by the authors, without undue reservation.
