# Supplementary material for: Comparative differences in job preferences among doctors in different levels of public hospitals in Henan, China: a discrete choice experiment
Source: Front Psychol. 2025 Jul 23;16:1607061. doi: 10.3389/fpsyg.2025.1607061 (PMC12325187; doi:10.3389/fpsyg.2025.1607061)
Supplement: Supplementary file 1 [file Supplementary_file_1.docx]

**Appendix 1 The attributes related job preference from reviewing literature**

| **Attributes** | **Studies** |
| --- | --- |
| Monthly salary/monthly salary and allowance/Monthly income | ( Cui et al., 2023; İşlek & Şahin, 2023; Lamba et al., 2021; Liu et al., 2022; Mumbauer et al., 2021; Okoroafor et al., 2021; Rao et al., 2013; Shiratori et al., 2016; Song et al., 2015; J. Wang et al., 2021; Wu et al., 2022; Yan et al., 2014) |
| Work location/Location | (Bao et al., 2023; Liu et al., 2022; Mumbauer et al., 2021; Rao et al., 2013; J. Wang et al., 2021; Yan et al., 2014) |
| Hospital level/Type of health center/Sector and Facility type | (Mumbauer et al., 2021; Rao et al., 2013; Song et al., 2015) |
| Training opportunities / Opportunities for training | (Lamba et al., 2021; Mumbauer et al., 2021; Yan et al., 2014) |
| Career promotion speed | (Liu et al., 2022; Okoroafor et al., 2021; Yan et al., 2014) |
| Career development/Career opportunity/Opportunities of professional development | (Cui et al., 2023; Gautam et al., 2019; İşlek & Şahin, 2023; Rao et al., 2013; Song et al., 2015; J. Wang et al., 2021; P. Wu et al., 2022) |
| Educational opportunities | (Bao et al., 2023; İşlek & Şahin, 2023) |
| Work environments/work condition/Infrastructure, equipment, supplies/work equipment/ Infrastructure/Essential equipment/Availability of Equipment | (Bao et al., 2023; Cui et al., 2023; İşlek & Şahin, 2023; Lamba et al., 2021; Liu et al., 2022; Mumbauer et al., 2021; Okoroafor et al., 2021; Rao et al., 2013; Shiratori et al., 2016; Song et al., 2015) |
| Availability of technological support | (Gautam et al., 2019) |
| Work stress/workload | (Angell et al., 2021; Bao et al., 2023; İşlek & Şahin, 2023; Lamba et al., 2021; Mumbauer et al., 2021; J. Wang et al., 2021; P. Wu et al., 2022) |
| Workplace Culture/Team in the job setting | (Cui et al., 2023; Gautam et al., 2019; Mumbauer et al., 2021) |
| Safety at workplace | (Angell et al., 2021) |
| Doctor–patient relationship | (Bao et al., 2023) |
| Rewards and punishments/Career path based on attendance/Policies to address absenteeism | (Angell et al., 2021) |
| Management style | (Cui et al., 2023; Lamba et al., 2021; Shiratori et al., 2016) |
| Years of work before study leave | (Shiratori et al., 2016) |
| Educational condition for children/Provision of education for children /allowance for children’s education/ Creche | (Gautam et al., 2019; İşlek & Şahin, 2023; Liu et al., 2022; Shiratori et al., 2016; Yan et al., 2014) |
| Housing/Providing house/allowance provided,government housing with amenities | (İşlek & Şahin, 2023; Liu et al., 2022; Okoroafor et al., 2021; Shiratori et al., 2016) |
| Transportation/ Provision of car allowance | (Bao et al., 2023; Gautam et al., 2019; Shiratori et al., 2016) |
| Benefits/Welfare benefits/ | (Mumbauer et al., 2021; Song et al., 2015) |
| Bianzhi | (Bao et al., 2023; Cui et al., 2023; Liu et al., 2022; Yan et al., 2014) |
| Opportunity for private practice | (Gautam et al., 2019) |
| Relationship between the doctor and the local community | (Angell et al., 2021) |
| Respect from society/Respect from the community | (Song et al., 2015; Wu et al., 2022) |
